# Retraction: An Aspirated Tooth Masquerading As Lung Cancer: A Unique Case Report

**DOI:** 10.7759/cureus.r141

**Published:** 2024-04-30

**Authors:** Emmanuel Meram, Meghan Mansour, Ali Khreisat, Roa'a AlKloub, Bhavinkumar Dalal

**Affiliations:** 1 Internal Medicine, Wayne State University School of Medicine, Detroit, USA; 2 Internal Medicine, Oakland University William Beaumont School of Medicine, Rochester Hills, USA; 3 Internal Medicine, Corewell Health William Beaumont University Hospital, Royal Oak, USA; 4 Pulmonary Critical Care Medicine, Corewell Health William Beaumont University Hospital, Royal Oak, USA

This article has been retracted by the Editor-in-Chief due to conflicting claims of authorship that cannot be adjudicated. The authors have requested retraction to avoid further issues and, after careful consideration, the journal has agreed to retract. In addition, there is one key scientific error in the article as the aspirated material was a tooth filling, not an actual tooth as claimed.

